# Effect of the Level of Anesthesia on the Auditory Brainstem Response in the Emei Music Frog (*Babina daunchina*)

**DOI:** 10.1371/journal.pone.0169449

**Published:** 2017-01-05

**Authors:** Jianguo Cui, Bicheng Zhu, Guangzhan Fang, Ed Smith, Steven E. Brauth, Yezhong Tang

**Affiliations:** 1 Chengdu Institute of Biology, Chinese Academy of Sciences, Chengdu, Sichuan, China; 2 Department of Psychology, University of Maryland, College Park, MD, United States of America; Universidad de Salamanca, SPAIN

## Abstract

Anesthesia is known to affect the auditory brainstem response (ABR) in mice, rats, birds and lizards. The present study investigated how the level of anesthesia affects ABR recordings in an amphibian species, *Babina daunchina*. To do this, we compared ABRs evoked by tone pip stimuli recorded from 35 frogs when Tricaine methane sulphonate (MS-222) anesthetic immersion times varied from 0, 5 and 10 minutes after anesthesia induction at sound frequencies between 0.5 and 6 kHz. ABR thresholds increased significantly with immersion time across the 0.5 kHz to 2.5 kHz frequency range, which is the most sensitive frequency range for hearing and the main frequency range of male calls. There were no significant differences for anesthetic levels across the 3 kHz to 6 kHz range. ABR latency was significantly longer in the 10 min group than in the 0 and 5 min groups at frequencies of 0.5, 1.0, 1.5, 2.5 kHz, while ABR latency did not differ across the 3 kHz to 4 kHz range and at 2.0 kHz. Taken together, these results show that the level of anesthesia affects the amplitude, threshold and latency of ABRs in frogs.

## Introduction

Many studies have investigated acoustic communication in anuran species [1–4]; however, fewer have used electrophysiological methods to investigate hearing sensitivity in these species [2, 5–8]. Nevertheless, hearing sensitivity and vocalization characteristics are generally thought to co-evolve insofar as the signal characteristics and receiver sensitivity to some of those characteristics would be expected to match. The auditory brainstem response (ABR) provides a good estimate of the shape of the behavioral audiogram [5, 9–12] and is thus an extremely useful tool for studying hearing sensitivity as well as the functionality of the auditory system. ABR morphology is similar across most vertebrate classes [13–15] consisting of a series of voltage peaks occurring within the first few milliseconds (ms) after the onset of an auditory stimulus and representing the progressive propagation of neural activity through the ascending auditory pathway.

Experimental electrophysiological recordings of the auditory system are frequently performed under anesthesia. General anesthesia is necessary to prevent movement of the animal to minimize artifacts during recording. Anesthesia is known to affect the auditory brainstem response in some species including mice, rats and lizards [15–20]. However, there is no report concerning the effects of the level of anesthesia on auditory brainstem responses in amphibians, the most important vertebrate transition from water to land. Tricaine methane sulphonate (MS-222) is an anesthetic commonly used in electrophysiological recording experiments in aquatic organisms such as fish [21] and anurans [22–23] because it is easily absorbed by immersion in the water bath. In the present study, we compared the effects of different levels of MS-222 on ABRs elicited by tone pip stimuli in the Emei music frog (*Babina daunchina*). This species was chosen because many previous studies have investigated acoustic communication and hearing in *B*. *daunchina* using both behavioral and electrophysiological methods [24–29]. These studies show that the most sensitive frequency range for hearing and the main frequency range of male calls is 0.5–3 kHz [26, 28, 30].

## Materials and Methods

### Ethics Statement

All applicable international, national, and/or institutional guidelines for the care and use of animals were followed. All procedures performed in studies involving animals were approved by the Animal Care and Use Committee of Chengdu Institute of Biology, CAS (CIB2013041501). The frogs were used in the experiments with the permission of the management office of the Mt. Emei nature reserve. This article does not contain any studies with human participants performed by any of the authors.

### Animals and anesthetic level treatment

Experiments were conducted from May to August, 2013. Sixteen male and nineteen female adult Emei music frogs were captured from ponds on Mt. Emei (29.36° N, 103.22° E). The frogs were maintained in tanks containing water and aquatic plants with natural temperature and photoperiod. The temperature of the water and room air was maintained at 23 ± 1°C. The subjects were fed live crickets and individually marked with passive integrated transponder (PIT) tags (Hongteng, Inc. China). The 0.2% solution of MS-222 was freshly prepared in water taken from the animal’s original pond.

The subjects were anesthetized via water immersion with 0.2% solution of MS-222. We monitored the level of anesthesia by testing the animal’s response to a gentle pinch to a toe-tip once per 15 s. The time point at which subjects failed to respond to a gentle toe pinch was defined as 0 minutes after anesthesia induction. The level of anesthesia was varied by increasing the duration of the MS-222 immersion period before commencing ABR recording at 0 minutes, 5 minutes or 10 minutes after the subject failed to respond to a gentle pinch to a toe-tip. We also recorded the amount of time it took for an animal to become anesthetized (Induction time) and the time the subject fully awakened from anesthesia after ABR measurement (Recovery time). After fully awakening, the animals were returned to the tanks and fed crickets until ABRs were recorded again. Each subject was recorded three times for each of three different anesthetic levels (i.e. 0, 5, 10 minutes after the subjects failed to respond to a pinch to a toe-tip). The interval between successive ABR recordings was 48 hours and the order of the three different anesthetic level treatments was randomized.

### ABRs measurements

ABRs were recorded in a soundproofed acoustic chamber (0.5 × 0.5 × 0.5 m). The temperature of the chamber was maintained at 23 ± 1°C. The stimulus presentations, ABR acquisition, equipment control, and data management have been described previously [28, 31]. For each subject three 27 gauge stainless steel electrodes (Rochester Electro-Medical, Inc. FL, USA) were inserted subdermally, at the midline above the medulla (about 3 cm caudal to the snout), above the tympanum and in the ipsilateral front leg as inverting, noninverting and ground electrodes, respectively. The recording electrodes were connected to a head stage and amplifier (PA4 & RA4, 20x gain, TDT) via wires wrapped in aluminum foil.

Stimulus generation and ABR recording were carried out using a digital signal processor RM2 (Tucker-Davis Technologies, Gainesville, USA), via fiber optic cables linked to the RA4 and a USB linked to a laptop computer running custom software (Open ABR) developed by Ed Smith (The University of Maryland, USA) based on Matlab 2009a (The MathWorks, Inc. USA). Two types of stimuli, tone pips and spectrally broadband clicks (as control), were generated by Open ABR and delivered through a portable amplified field speaker (SME-AFS, Saul Mineroff Electronic Inc, USA) driven by the RM2 and positioned in front of the frog’s head. Stimuli were synthesized digitally from 0.5 kHz to 6.0 kHz, in 0.5 kHz intervals with a stimulation duration of 1 ms rise/fall time (linear ramp), 3 ms plateau time and sample rate of 24414 Hz. The 0.01 ms broadband clicks were synthesized and used as stimuli to verify the presence of a biological signal in response to sound and to verify that neural responses to sound did not change during a recording session. Visual inspection of these click-evoked ABRs indicated there were no response changes over the duration of each recording session.

Prior to ABR recording, the sound pressure levels of all the stimuli were calibrated using a G.R.A.S. 46BE 1/4 inch microphone (G.R.A.S. Sound & Vibration, Denmark) with CCP Supply (Type 12AL, G.R.A.S. Sound & Vibration, Denmark) positioned at the location of the frog’s head (10 cm from the speaker). ABR stimuli were delivered in random order with the frequency varying from 0.5 kHz to 6.0 kHz. The presentation rate was 4/s. For all stimuli, we obtained two replicate averages of the ABR, each based on averaging responses to 400 consecutive presentations of each stimulus train (800 presentations total across both replicates). The polarity of the stimulus waveform is alternated. We presented the entire set of stimulus frequencies before the second replication. Stimuli were initiated at 45 dB SPL and increased in 5 dB steps based on the assumption that all ABR thresholds in this species are below 90 dB. All biological signals were notch filtered at 50 Hz during data collection.

ABR thresholds and latencies were determined using methods described in previous studies [28, 31]. Threshold measurements were defined as the lowest stimulus level for which no responses could be recognized by two experienced observers independently by visual inspection. The mean value of the two observers was used for the analysis. ABR latencies were measured between stimulus onset and the first ABR waveform valley using manually placed cursors in Matlab (Version 2009a).

### Analysis and statistics

ABR morphologies, thresholds and latencies obtained from male and female Emei music frogs in response to tone pips were sorted and analyzed using SigmaPlot 11 software (Systat Software Inc., San Jose, USA). All data were examined for assumptions of normality and homogeneity of variance, using Shapiro-Wilk and Levene tests, respectively. The differences in ABR thresholds and latency among different anesthetic levels were assessed using three Way Analysis of Variance (ANOVA) (anesthetic level, sex and frequency as factors), followed by the Holm-Sidak post-hoc test. The differences in induction and recovery times for the different anesthetic levels were assessed using two way repeated measures ANOVA (anesthetic level and sex as factors). For all tests, data were expressed as Mean ± SD; P < 0.05 was considered to be statistically significant.

## Results

### ABR wave morphology

**Fig 1:**
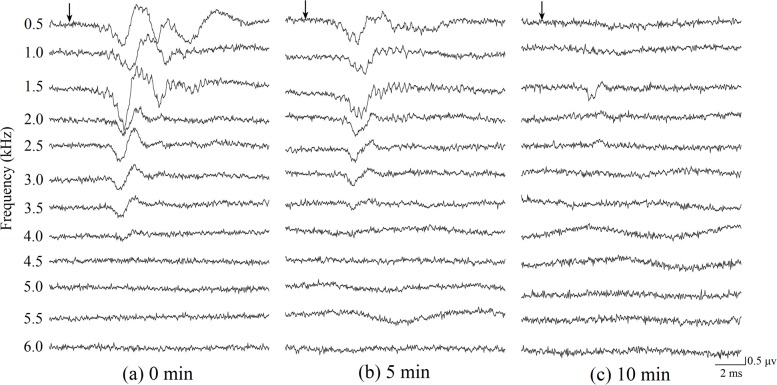
**ABR replicates elicited in response to frequency-specific tone pips at 70 dB SPL showing valley-peak waveforms for three different anesthesia levels recorded from the same frog, (a) 0 minutes after anesthesia induction, (b) 5 minutes after anesthesia induction and (c) 10 minutes after anesthesia induction.** Downward pointing arrows depict the arrival time of sound at the tympanic membrane.

Typical ABRs to tone pips for the three anesthetic levels showed valley-peak waveforms occurring within 4–7 ms of the stimulus onset (Fig 1). The dominant valleys and peaks of the ABR in response to 0.5–4 kHz tone pips at 70 dB SPL were easily visible in the 0 minutes group (Fig 1). Although ABRs obtained in the 10 minutes group did not always show distinctive valleys and peaks, ABR wave morphologies were similar for the 0 min and 5 min anesthetic groups (Fig 1).

### ABR thresholds

**Fig 2:**
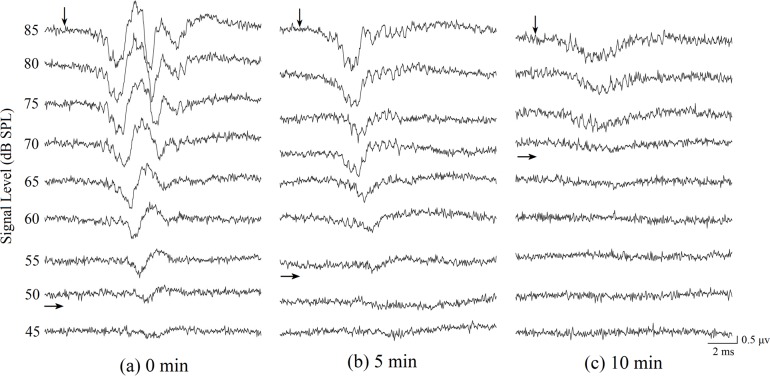
**ABR waveforms as a function of stimulus intensity evoked by 1 kHz tone pips for three different anesthesia levels obtained from the same frog, (a) 0 minutes after anesthesia induction, (b) 5 minutes after anesthesia induction and (c) 10 minutes after anesthesia induction.** Downward pointing arrows depict the arrival time of sound at the tympanic membrane. The right-pointing arrowheads depict the visually detected thresholds for each anesthesia level.

**Fig 3:**
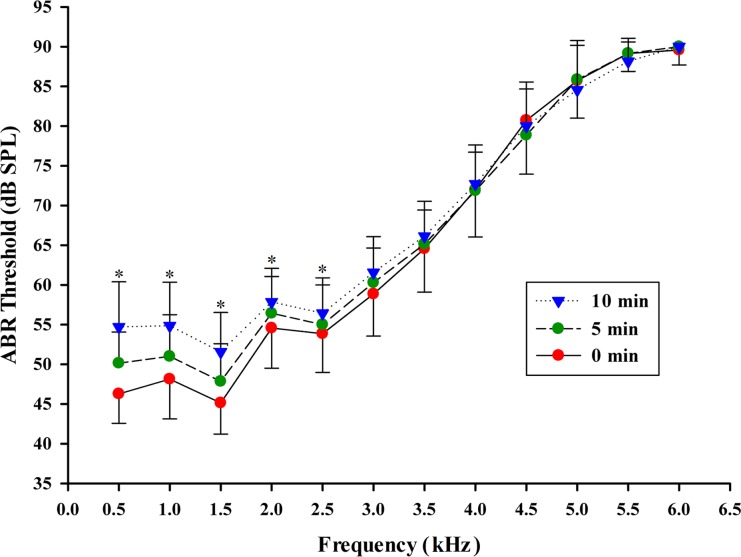
ABR thresholds for Emei music frogs for three different anesthesia levels recorded 0 minutes after anesthesia induction, 5 minutes after anesthesia induction and 10 minutes after anesthesia induction. The points plotted represent the thresholds for tone pips (Mean ± SD). Asterisks indicate significant differences between groups (p < 0.05).

ABR level series were obtained for threshold estimation from each animal at frequencies between 0.5 and 6 kHz for the three anesthetic levels (Fig 1). Fig 2 shows a typical ABR level series in response to 1 kHz tone pip stimuli, measured for the three anesthetic levels (Fig 1 and Fig 2 were recorded from the same frog). As can be seen in this figure, ABR thresholds increased from 50, to 55, and to 70 dB SPL as the anesthetic level increased. The amplitude modulated waveforms evoked by tone pip are shown in Fig 1 and Fig 2. As shown, ABR amplitudes visibly decrease with immersion anesthesia time. These results are summarized quantitatively in Fig 3, which shows ABR threshold as a function of tone pip frequency for the three anesthetic conditions.

The effects of anesthetic level, sex and frequency on ABR thresholds were analyzed using three Way Measures ANOVA. There are significant differences in ABR thresholds between different anesthetic levels (F = 31.979, P < 0.001) and frequencies (F = 1922.980, P < 0 .001) while sex has no significant effect on the ABR thresholds (F = 0.967, P = 0.326). There is no statistically significant interaction effect between anesthetic levels and sex (F = 0.803, P = 0.448). For this reason ABR threshold data from male and female subjects were combined for subsequent analysis (Fig 3). The effect of the level of anesthesia depends on stimulus frequency. There is a statistically significant interaction between anesthetic levels and frequency (F = 4.984, P < 0.001). The results show that ABR thresholds significantly increase with increasing immersion time across the 0.5 kHz to 2.5 kHz frequency range, which is the most sensitive frequency range of the auditory system and the main frequency range of male calls. In contrast ABR thresholds are not significantly different between the three anesthetic levels across the 3 kHz to 6 kHz range (Fig 3). There is a statistically significant interaction between sex and frequency (F = 4.486, P < 0.001) with significant sex differences at 2 kHz (P = 0.011), 2.5 kHz (P < 0.001), 4.5 kHz (P < 0.001) and 5 kHz (P < 0.001). No significant sex differences were found for other frequencies (P > 0.05).

### ABR latencies

**Fig 4:**
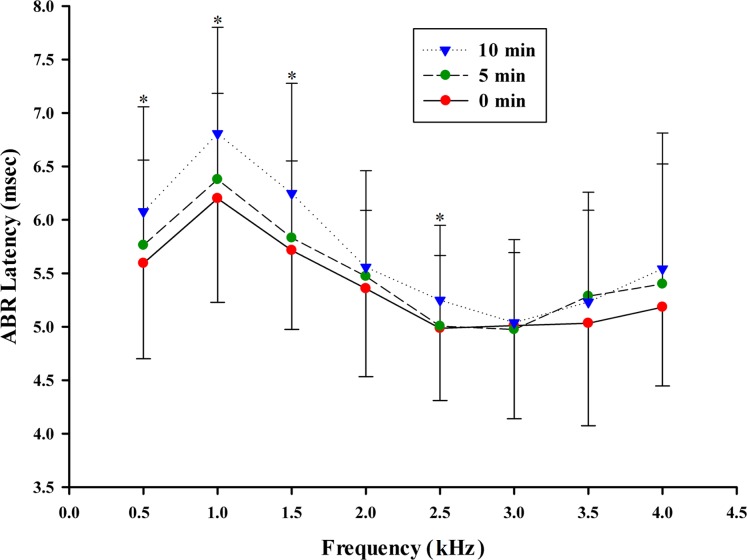
ABR latency to valley for each frequency at 70 dB SPL. (Mean ± SD, n = 35). Asterisks indicate significant differences between groups (p < 0.05).

ABR latency is an additional indicator of auditory sensitivity. ABR latencies were measured between stimulus onset and the waveform valley (Fig 1, Fig 2) (note: the time of ABR latencies contain the acoustic delay from the speaker to the frog which is about 0.29 ms). Latencies were analyzed for tone pip frequency at 70 dB SPL, in accordance with the fact that ABR latencies typically become shorter as stimulation intensities increase [32]. The latency analysis was only performed from 0.5 kHz to 4.0 kHz due to the fact that thresholds for the 4.5 to 6 kHz range were above 70 dB; thus there were no significant responses in the 4.5 kHz to 6 kHz range to the 70 dB stimulus. There are significant differences in ABR latency for different anesthetic levels (F = 12.284, P < 0.001), sex (F = 158.816, P < 0.001) and frequencies (F = 40.454, P < 0 .001). There is no statistically significant interaction between anesthetic levels and sex (F = 1.175, P = 0.309), between anesthetic levels and frequency (F = 0.746, P = 0.729) and between sex and frequency (F = 1.032, P = 0.407). The results show that ABR latency is significantly longer in the 10 min group than in the 0 and 5 min group at 0.5, 1.0, 1.5 and 2.5 kHz (p < 0.05, Fig 4). There were no significant ABR latency differences between the three anesthetic levels for frequencies of 2.0, 3, 3.5 and 4 kHz (p > 0.05, Fig 4).

### Induction and Recovery Times

There was no significant difference in induction time among the three anesthesia groups (6.6 ± 1.0 min for 0 min group, 7.3 ± 1.0 min for 5 min group, 6.8 ± 0.9 min for 10 min group, F = 1.364, P = 0.267) and between sex (F = 3.486, P = 0.068). In contrast recovery time (the time for subjects to fully awaken from anesthesia after ABR measurement) was significantly increased by increasing immersion time (56.7 ± 34.5 min for 0 min group, 70.8 ± 29.3 min for 5 min group, 122.1 ± 44.0 min for 10 min group, F = 49.005, P < 0.001). Sex did not have a significant effect on recovery time (F = 0.00410, P = 0.949). There is no significant interaction between anesthetic levels and sex in recovery time (F = 0.449, P = 0.640). The duration of the ABR recordings are similar for the three groups (about 68 min).

## Discussion

Previous studies have shown that for humans and most animals, the ABR is not affected by sleep and/or sedation [33–34]. Nevertheless the ABR is affected by anesthesia in some animals including mice, rats and lizards [15–20]. Furthermore, previous studies have not looked at the effects of the duration or level of anesthesia on the ABR in fish or frogs. The present study shows that in one amphibian species, the Emei music frog (*Babina daunchina*), anesthetic effects on ABR exist.

MS-222 is an anesthetic commonly used in aquatic organisms such as in fish and anurans during ABR measurement [28, 35–38]. The current study was undertaken to address the issue of whether the duration of MS-222 anesthesia affects repeated measures studies of the auditory system. If MS-222 anesthesia duration has a significant effect on ABR results, it would render invalid comparisons between any data collected from subjects exposed to anesthesia for different periods of time following induction.

In the present study, effects of anesthetic level, sex and frequency on ABR thresholds and latency was investigated in Emei music frog (*B*. *daunchina*). The waveform shape is similar with that of Cope’s gray treefrog (*Hyla chrysoscelis*) [8] and (Xenopus laevis) [36].The ABR latency is also very similar to that of Cope’s gray treefrog (*Hyla chrysoscelis*), for which the longest latency is 5.5ms to 6.5ms at 1 kHz to 1.5 kHz. In the present study, the shape of the ABR audiograms in the Emei music frog (*B*. *daunchina*) did not change as a function of anesthesia level and showed similar peak sensitivity across the 0.5 kHz to 2.5 kHz frequency range [28]. However, anesthesia level does affect ABR thresholds, latencies and amplitudes in this species. Thus, although the ABR wave morphology was similar across the treatments, ABR thresholds significantly increased with immersion time across the 0.5 kHz to 2.5 kHz frequency range, the most sensitive frequency range for hearing [28], while anesthesia had little effect across the 3 kHz to 6 kHz range. Similarly, ABR latency was significantly longer in the 10 min group than in the 0 and 5 min groups at 0.5, 1.0, 1.5 and 2.5 kHz, while anesthesia did not affect latency for the 3 kHz to 4 kHz range and at 2.0 kHz. The differences in ABR thresholds and latency for the different stimulus frequency ranges may result from the differences in the mode of operation of the specialized amphibian inner ear consisting of the amphibian papillae (AP) and basilar papillae (BP) [39]. By selectively lesioning the eighth-nerve branchlets innervating individual sensory maculae within the otic capsule of the bullfrog inner ear, it has been demonstrated that fibers tuned to low- and mid-frequencies innervate the AP and fibers tuned to higher frequencies innervate the BP [2]. Nevertheless further study is needed to determine if the effects of MS-222 on the AP and BP are different. In Cope’s gray treefrog (*Hyla chrysoscelis*) [8], latencies were slightly shorter for females compared to males, particularly at tone frequencies below ~1.75 kHz, resulting in a significant main effect of sex and its interaction with frequency. For the Emei music frog, there is no statistically significant interaction between anesthetic levels and sex, between anesthetic levels and frequency and between sex and frequency, which may reflect species specific differences.

The results show that waveform amplitudes evoked by tone pips tend to decrease with immersion anesthesia time. These results are similar to those reported for mice showing that ABR thresholds are significantly lower in the awake than in the ketamine/xylazine anesthetized condition (difference of 8.0 ± 1.8 dB) [19]. In contrast varying isoflurane levels in geckos and anoles reveals little effect on the ABR. Changing the level from 3%, which produces full anesthesia, to 0.5%–1%, which lightly anesthetizes or sedates the subjects, produced no significant changes in sensitivity [15]. The present results reveal that MS-222 anesthesia may yield different effects in anurans either because of species differences or the nature of the anesthetic.

MS-222 is an anesthetic commonly used in aquatic organisms. The physiological effect of anesthetics is of great interest since these are frequently used in research and routine aquaculture procedures to immobilize fish and minimize stress responses [40]. MS-222 is absorbed through the skin and rapidly enters the blood stream in frogs. Higher concentrations of MS-222 result in more rapid anesthesia with shorter maximum tolerated exposure times, while lower concentrations of MS-222 result in longer induction times and longer maximum tolerated exposure times, and are recommended for maintenance of anesthesia [41]. In the present study, we used relatively low concentrations of MS-222 (0.2%) in the ABR experiments. For ABR studies it is important to maintain the subjects at a depth of anesthesia sufficient to immobilize them for the duration of the experiment yet not too deep to affect the ABR response or cause the subjects to die. The mechanism by which MS-222 acts as a muscle relaxant and anesthetic is through blocking voltage-gated sodium channels in the nerve membrane thereby reducing membrane excitability and the likelihood that action potentials are produced [42]. ABRs are the combined electrical response of the auditory nerve and brain nuclei, likely explaining why the level of anesthesia induced by MS-222 may affect the ABR, although these effects can vary between species as discussed below.

The action of MS-222 as an anesthetic varies widely between species and is affected by water temperature, dissolved minerals (i.e. water hardness), and the size of the individual animal [43]. It is difficult to measure the plasma concentration of MS-222 for monitoring the level of anesthesia during ABR measurement. Therefore we used the length of time after the subjects first failed to respond to a gentle toe-tip pinch to control the anesthesia level. It is important to be able to standardize the level of anesthesia during electrophysiological studies. Thus both induction time and recovery time were measured. There were no significant differences in induction time between the three anesthesia groups. In contrast, recovery time, (the time required for subjects to fully awaken from anesthesia after ABR measurement), was significantly increased by increasing immersion time. These results therefore show that immersion time significantly affects both the depth of anesthesia as reflected by ABR thresholds as well as recovery time.

Taken together, the present study shows that the anesthetic levels do affect the amplitude, threshold and latency of the ABRs in frogs. Our results suggest that investigators need to take anesthetic levels into account in ABR experiments in order to be able to compare results obtained in different studies or between different species.
